# Supplementary figures and images for: Morphological, molecular and MALDI-TOF MS identification of ticks and tick-associated pathogens in Vietnam
Source: PLoS Negl Trop Dis. 2021 Sep 28;15(9):e0009813. doi: 10.1371/journal.pntd.0009813 (PMC8500424; doi:10.1371/journal.pntd.0009813)

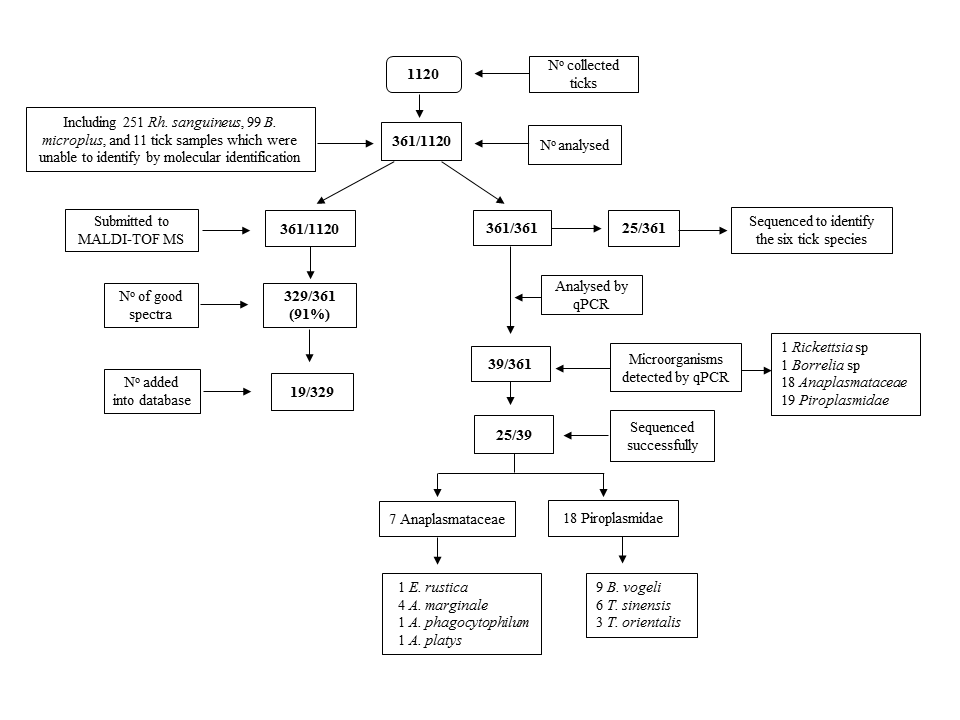

Supplement: S1 Fig — (TIF) [file pntd.0009813.s001.tif]
