# Supplementary material for: Morphological, molecular and MALDI-TOF MS identification of ticks and tick-associated pathogens in Vietnam
Source: PLoS Negl Trop Dis. 2021 Sep 28;15(9):e0009813. doi: 10.1371/journal.pntd.0009813 (PMC8500424; doi:10.1371/journal.pntd.0009813)
Supplement: S1 Table — *: Tick was co-infections by two microoganisms. (DOCX) [file pntd.0009813.s002.docx]

**S1 Table: The number of microorganisms were detecetd in engorged/non-engored ticks**

| **Tick species** | **Code** | **Microorganisms detected** | **Engorged** |
| --- | --- | --- | --- |
| *B. microplus* | 18 | *Ehrlichia rustica* | Yes |
| *B. microplus** | 20B | *Anaplasma.phagocytophilus, Theileria sinensis* | Yes |
| *B. microplus* | 20A | *Theileria sinensis* | Yes |
| *Rh. sanguineus* | 15 | *Babesia vogeli* | No |
| *Rh. sanguineus* | 98 | *Babesia vogeli* | Yes |
| *B. microplus* | 351 | *Theileria sinensis* | Yes |
| *B. microplus* | 357 | *Theileria sinensis* | Yes |
| *B. microplus* | V6-30 | *Theileria sinensis* | Yes |
| *B. microplus* | V6-31 | *Theileria sinensis* | Yes |
| *Rh. sanguineus* | 189 | *Babesia vogeli* | No |
| *Rh. sanguineus* | 190 | *Babesia vogeli* | No |
| *Rh. sanguineus* | 242 | *Babesia vogeli* | No |
| *Rh. sanguineus* | 247 | *Babesia vogeli* | No |
| *Rh. sanguineus* | 252 | *Babesia vogeli* | No |
| *Rh. sanguineus* | 267 | *Babesia vogeli* | No |
| *Rh. sanguineus* | 286 | *Babesia vogeli* | No |
| *B. microplus* | D28-139 | *Theileria orientalis* | Yes |
| *B. microplus* | D28-140 | *Theileria orientalis* | Yes |
| *B. microplus* | D28-141 | *Theileria orientalis* | Yes |
| *B. microplus* | B24-94A | *Anaplasma marginale* | Yes |
| *B. microplus* | B24-98 | *Anaplasma marginale* | Yes |
| *B. microplus* | V-106 | *Anaplasma marginale* | Yes |
| *Rh. sanguineus* | Q-134 | *Anaplasma marginale* | Yes |
| *Rh. sanguineus* | Q-137 | *Anaplasma platys* | Yes |

*: Tick was co-infections by two microoganisms
