# Supplementary material for: Morphological, molecular and MALDI-TOF MS identification of ticks and tick-associated pathogens in Vietnam
Source: PLoS Negl Trop Dis. 2021 Sep 28;15(9):e0009813. doi: 10.1371/journal.pntd.0009813 (PMC8500424; doi:10.1371/journal.pntd.0009813)
Supplement: Attachment — Submitted filename: renamed_63381.pdf [file pntd.0009813.s003.pdf]

Certificate of translation according to "ISO 17100:2015" standard

The "ISO 17100:2015" quality standard is the specific European standard for translation services; it describes the recommended translation process, as well as related aspects regarding the delivery (including quality assurance and traceability).
Translation process: Translation by a professional translator, followed by a proofreading by another professional translator.
Qualification of the translation team:
The translators and proofreaders involved in this project comply with ISO 17100:2015. They have:

- *A postgraduate degree in translation,*
- *More than five years of experience in translation.*
- *They all worked towards their mother tongue, and are experts in the field of translation*

**Documents to be translated**			**Languages**	
**Description**	**Version** (If Applicable)	**Date** (If Applicable)	**From**	**To**
Morphological, molecular and MALDI-TOF MS identification of ticks and tick-associated pathogens in Vietnam				**English**

Translator

I, the undersigned, certify that I have performed the translation(s) listed above.

Name	Signature	Date:
Employer	Position/Qualifications	

Reviewer (if applicable)

I, the undersigned, certify that I confirm the accuracy of the translations(s) listed above.

IM<br>Name	Signature	Date: **06/09/2021**
TRADONLINE<br>Employer	Position/Qualifications	

TRADONLINE SARL
Agence de Traduction
25 Rue du Douanier Rousseau - 53000 LAVAL
Tél. 02 43 53 52 41
E-mail : contact@tradonline.fr
SIRET 502 435 852 00048

TradOnline
More than translations
